# Developing inclusive public involvement and engagement activities *with* secondary school students and educational professionals: a protocol

**DOI:** 10.1186/s40900-024-00581-3

**Published:** 2024-07-01

**Authors:** Lauren Cross, Dale Banham, G. J. Melendez-Torres, Tamsin Ford, Esther van Sluijs, Kristin Liabo

**Affiliations:** 1 University of Cambridge (MRC Epidemiology Unit), Cambridge, UK; 2 Northgate High School, Ipswich, UK; 3 University of Exeter (Medical School), Exeter, UK; 4 Department of Psychiatry, University of Cambridge, Cambridge, UK

**Keywords:** Public involvement and engagement, Young people, Secondary schools, Diversity and inclusion, Protocol, Doctoral research

## Abstract

**Background:**

Public involvement and engagement (PI&E) is increasingly recognised as an important component of research. It can offer valuable insights from those with experiential knowledge to improve research quality, relevance, and reach. Similarly, schools are ever more common sites for health research and, more recently, PI&E. However, ‘gold-standard’ practice is yet to be established, and activities/approaches remain underreported. As a result, knowledge can remain localised or lost. Diversity and inclusion also remains a challenge.

**Methods:**

This protocol has been informed by UK national guidance, evidence-based frameworks and available implementation literature. It describes both rationale and approach to conducting PI&E activities within a secondary school context. Activities are designed to be engaging, safe and accessible to young people with diverse experiences, with scope to be iteratively developed in line with public collaborator preference.

**Discussion:**

Young people should be architects of their involvement and engagement. Ongoing appraisal and transparency of approaches to PI&E in school settings is crucial. Expected challenges of implementing this protocol include facilitating a safe space for the discussion of sensitive topics, absence and attrition, recruiting students with a diverse range of experiences, and potential knowledge and capacity barriers of both facilitator and contributors. Activities to mitigate these risks are suggested and explored.

## Abstract (plain language summary)

Schools are increasingly becoming hubs for health research. However, there is a lack of knowledge about how researchers, schools and students can best work together to shape the studies we do. This is a problem as, in the world of research, involving those with first hand experiences (public collaborators) in the research process is seen as crucial.

This protocol outlines our plan for conducting public involvement and engagement activities in secondary schools. It is based on national guidance and existing evidence. The goal is to make these activities interesting, safe, and accessible to young people with diverse experiences. The approach is designed to be flexible, allowing adjustments based on the preferences of the public collaborators.

We acknowledge that we may face some difficulties with our approach. This may include challenges in recruitment of public collaborators, dealing with absence and attrition, and creating a safe space for discussing sensitive topics. Collaborators from both academic and lived backgrounds may also experience barriers in knowledge and capacity. This protocol suggests activities to address and overcome these challenges. We emphasise the need for ongoing evaluation and transparency in public involvement and engagement approaches within school settings.

## Background

Use of public involvement and engagement within health research has gained considerable traction across the last two decades. It has the potential to offer insights from those with lived and relevant experiences to improve research quality, relevance, and reach of research [1, 2]. While some rationales emphasise moral obligations and the potential to promote epistemic justice through involvement [3], others highlight cautionary tales of wasted opportunity when stakeholders needs are not considered [4].

Recently, the WHO-UNICEF-Lancet launched a call for the involvement of young people in all decision-making [5]. However, although public involvement and engagement (PI&E) knowledge is rapidly evolving, literature is limited when it comes to describing best practice with adolescent populations [6]. Moreover, moving beyond tokenism, diversity of experiences, power imbalances, and accessibility remain key challenges for PI&E [7–10]. For example, in some cases, public contributors have been documented to feel inferior to researchers within PI&E spaces [11]. Whereas, Egbert and Nanna (2009) caution researchers against assuming health literacy [12]. This, alongside assumed methodological knowledge, can make dialogues inaccessible to those involved in PI&E, thereby limiting its potential.

There is considerable debate and definitional ambiguity surrounding the conceptualisation and nomenclature used within ‘involvement’ and ‘engagement’ literature [13, 14]. For the purpose of this protocol, the National Institute for Health and Care Research School for Public Health Research (NIHR SPHR) definition of PI&E will be adopted. This is founded in, and builds upon, both NIHR involve guidelines and the National Standards for Public Involvement definitions, terms and practices [15, 16]. The NIHR SPHR definition of PI&E makes a clear distinction between involvement and engagement. Involvement is considered “*research done in collaboration with or by the public and not to, about or for them”* [17]. Whereas, engagement with research involves “*sharing research findings and implications about research with members of the public to encourage dialogue and to share knowledge*” [17]. This means that the public should not only be (a) considered active agents within the research process but (b) provided access to key research knowledge.

‘Public’ is also a relatively broad, ambiguous term. This protocol considers people with relevant experience or those likely impacted by research findings as the ‘public’. Given the wider programme of research in which the activities described in this protocol are to be conducted, the primary public to involve are secondary school students. However, research participation within school settings also requires involvement and engagement of further stakeholders [18]. This includes, but is not exclusive to, educational professionals, parents/caregivers, researchers, and educational/health policymakers. Therefore, although we will mainly focus on the student perspective, opportunities to collaborate with further relevant groups will also be explored (see section on ‘supplementary PI&E’).

Young person public contributors will henceforth be referred to as *young person research advisors (advisors for short)*. This was established as appropriate terminology due to its clarity, and potential to be included within future Curricular Vitae and personal statements during early conversations with an educational professional (DB). Pairing this with university affiliation was deemed to further enhance the credibility and prestige of the opportunity.

### Context

The PI&E activities detailed within this protocol will be undertaken as part of a doctoral research project. Although commonly undertaken, descriptions and approaches to PI&E within doctoral contexts are rarely reported [19, 20]. The doctoral research project aims to explore young people’s participation in school-based health research, adopting a mixed-methods approach. Participation refers to participants taking part in a research study, and is distinct from involvement and engagement where individuals are actively involved within the research process- either through the design or sharing of results [21]. The research project will identify who is currently under-represented in school-based research studies and examine barriers and facilitators to participation in school-based health research. Given this context, involving a range of student voices to guide the project is essential. This will include gaining the perspectives of all eligible young people, including those from well represented and under-represented backgrounds within health research.

This protocol sets out the rationale, vision, and approach to embedding inclusive and accessible PI&E. It underpins the researchers’ commitment to actively involve young people as valued experts and research partners. This will enable us to purposefully, safely, and respectfully engage young people within the research process.

Our objectives include:

1. Produce research informed by, and relevant to, young people’s experiences, knowledge, and perceptions.
2. Produce research which is accessible and shared with key stakeholders, including young people.
3. Facilitate positively viewed experiences and skill/knowledge development for all collaborators.

## Methods

### Approach and guidance

All PI&E activities we will conduct will be shaped by existing, evidence-informed frameworks and national guidance. This includes the NIHR SPHR PI&E strategic guidance [17], NIHR INVOLVE guidance [15], UK Standards for public involvement [16] and Oliver et al’s (2015) framework for PI&E [22]. Although these are not targeted specifically to adolescent populations or school contexts, it was thought that there was sufficient transferability. In addition, implementation literature and practical ‘lessons learned’ will be drawn upon to help translate guidance into ‘what works’ in practice. This is important as both values and practicalities contribute to purposeful PI&E [23]. However, educational settings are complex environments to integrate PI&E within [18]. Most have long established expectations, policies and cultures. Where necessity has caused this protocol to deviate from established guidance this will be highlighted and tensions discussed.

### Framework

**Fig. 1:**
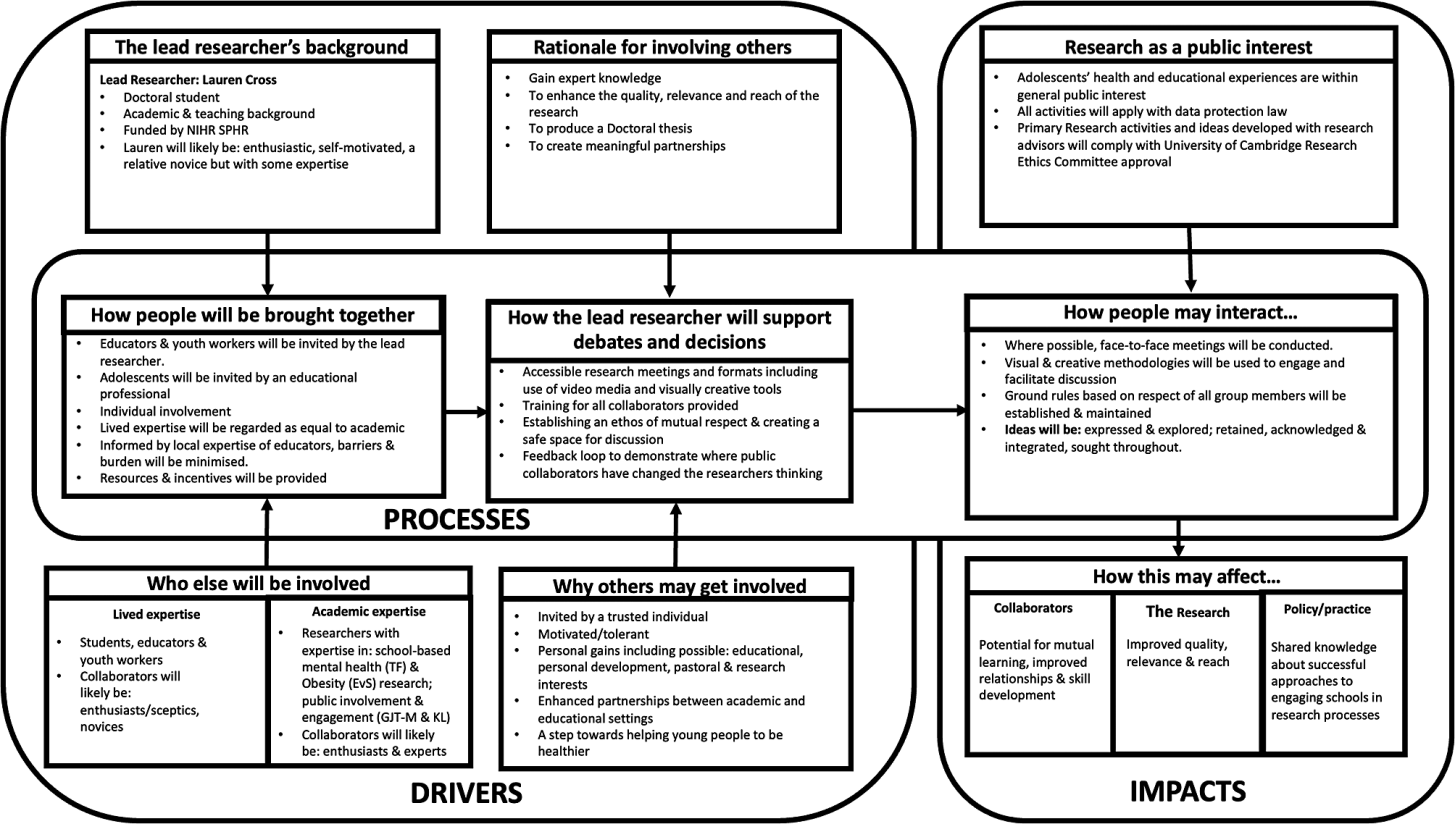
Application of Oliver et al’s framework to the present study

Oliver et al’s (2015) framework was developed with a view to inform the planning, organisation and evaluation of PI&E activities [22]. Classified as a power-focused framework [24], it advances traditional ladder approaches of participation (e.g. Arnstein, 1969 [25]) to also consider contextual factors. This includes consideration of different actors, processes, motivations, timing, and impacts for involvement/engagement in research. As a dynamic and flexible tool, Oliver et al’s framework was deemed an appropriate choice. It has also helped draw a focus to the specific needs and experiences of advisors during protocol development. For application of Oliver et al’s framework within this present study, please see Fig. 1.

### Recruitment and target group for involvement

Advisors will be invited through a mainstream, state-funded secondary school setting within the East of England. To address diversity of background/experience gaps often associated with PI&E groups, young people from a range of backgrounds will be invited to take part. Utilising local expertise of a senior educational professional (DB), approximately six students with different personalities, interests, and demographic, academic, and health backgrounds will be invited to participate. This will likely include those from different socio-economic positions, and gender and ethnic identities. Whilst some advisors will have lived experiences of living with physical and/or mental health conditions, others will not. Including individuals across a health spectrum was deemed important as school-based health research typically targets whole year/school cohorts.

The lead researcher (LC) had initially suggested working with 2–3 students; however, this was increased to ∼ 6 on the advice of senior school staff (DB). This will mean sessions are less vulnerable to the effects of absence and attrition. To minimise disruption of exam schedules, Year 10 students (ages 14–15) were suggested as an appropriate age group to initially participate (all UK students take standardised, national secondary school exams in Year 11).

The lead researcher (LC) will create and prepare targeted recruitment materials (including a brief flyer and information video). All contact and recruitment will be led by senior staff member (DB), who has also been central to the development of this protocol. The senior staff member will approach young people and ask if they are willing to become involved. The senior staff member has an established relationship of trust with the students. This approach will likely enhance participation as the invitation is coming from a highly-credible source [26].

Developing a transparent, and trusting partnership with the senior staff member (DB) has been essential to the development of our recruitment approaches and PI&E protocols. We hope this approach will help to ensure diversity of experience within our PI&E. Key to success was early and clear communication, founded on mutual respect and principles of reciprocity.

Although individual demographic details will not be collected, once recruited, the lead researcher will work with the school setting and advisors themselves to characterise group demographics. For example, aggregate details of group characteristics will be sought from routinely collected data from the host school. Any data provided by the school will comply with General Data Protection act (2018) Regulations and statistical disclosure guidelines. In addition, the lead researcher will work with advisors to co-develop a group description which captures demographics and experiences deemed relevant and important by advisors for the purpose of reporting and dissemination.

### Description of PI&E sessions

Watson et al. (2023) highlight that involving young people in all stages of decision-making creates optimal conditions to create opportunities and respect [27].

Advisors will therefore provide ongoing advice throughout the project from development of initial protocols to analysis and dissemination of results, through a number of advisory group sessions. Each session will last approximately one hour and take place within the school day. Sessions will be designed to be engaging, safe and training provided where appropriate (see below for further detail). To minimise participation barriers and participant burden [1], advisory group sessions will be conducted within school settings in the first instance. In conversation with a senior staff member (DB), the school conference room was identified as an appropriate location. This is a setting students would not typically inhabit, providing a sense of separation between every-day school and advisory activities. Furthermore, sessions will be designed to fit with school schedules, whilst also capturing key points within the project. In particular, sessions will be timetabled to avoid conflicts with school holidays and national exams. Sessions will occur more intensively at the start of the project to build momentum and enhance buy-in (occurring approximately once every three weeks). They will then decrease in frequency once the project is more established to further minimise burden/disruption (occurring approximately once every six-to-eight weeks).

### Promoting access and engagement

It has long been established that creative methods can be useful techniques for both the translation and production of knowledge [28]. More recently, Broomfield et al. (2021) highlight their ability to facilitate contributions from group members with varying needs, and even promote reflection [29]. Adopting varied, creative methodologies should, therefore, enable expression whilst capturing attention. However, building confidence will also be important. Utilising collaborative learning strategies within PI&E sessions should further motivate students to engage and express their ideas [30].

Furthermore, understanding of technical language is an established access barrier [12]. Due to the age and novice status of our advisors, it is unlikely they will already possess an understanding of health research techniques, concepts and terminology. Therefore, all technical aspects of the project will be translated into plain English at an appropriate literacy/academic level and delivered using evidence-informed pedagogy. This will include the use of scaffolding techniques [31], spiral curriculum [32] and spaced learning concepts [33].

Moreover, recent statistics highlight that 17% of young people in England have a Special Educational Need (SEN) [34]. Therefore, although individuals will not be singled out, additional consideration will be given to the meet the unique needs of those with SEN, with approaches informed by established best practice [35]. The lead researcher (LC) is also a qualified and experienced teacher, so will draw on her professional knowledge and existing skills to devise and develop appropriate content. Where appropriate, additional consultation will be sought from the senior educational professional (DB). This will ensure sessions are appropriately differentiated so *all* advisors can access the necessary content to engage in purposeful discussion. In addition, bespoke training opportunities will be provided (see next section for further details).

### Training

Lack of adequate training has been identified as a further barrier for public contributors participating in PI&E [10]. Similarly, the role of PI&E facilitator can be complex and requires a broad skillset. For example, Todd et al’s (2020) qualitative exploration highlights the multiple identities of the PI&E facilitator including the gatekeeper, negotiator and mediator [36]. Given the context of discussing potentially sensitive topics with novice research advisors, and a relatively novice lead researcher (LC), training and capacity building for all collaborators will form a central component of this protocol.

Under the advice of PI&E experts, the lead researcher (LC) will undertake formal and informal activities to develop her skills, competence, and confidence. This includes developing both theoretical and practical knowledge of implementing PI&E. This will likely involve attendance at carefully identified intensive specialist workshops and training sessions delivered by PI&E experts, including lived experience researchers. Additional training will also be delivered within doctoral supervisions.

In addition, all advisors will receive training on relevant research concepts, terminology, and techniques. This will mainly be delivered by LC and developed in response to project and individual needs. This may differ from person to person, as each collaborator’s skillset and experiences will be unique [37]. However, where training needs may reflect a more specialist skillset, additional external support will be sought. Such specialist training by external providers has been costed and will be conducted on a needs-basis. This may include the involvement of PI&E specialists and/or lived experience researchers as appropriate. Attendance at additional sessions will be carefully discussed with young people, and permission from both school and parents/caregivers sought. A brief introduction to PI&E will also be provided, with an emphasis drawn to the expert status of group members through their experiences.

### Creating a safe space

Creating a safe space to share experiences is essential. This is because PI&E collaborators are typically asked to share personal, potentially sensitive, information [1, 38]. Within this protocol, advisors will be considered an extension of the doctoral co-supervisory team in an attempt to subvert traditional power dynamics and hierarchies associated with both PI&E and adult-child relationships within school settings [11, 39]. Their viewpoints and advice will thus be treated with the same significance as a traditional doctoral supervisor, offering lived expertise to supplement the academic perspective. This will facilitate sharing of different knowledge types [3, 23].

Building quality, reciprocal partnerships can further help to mitigate potential power tensions [23, 40]. In practice, this will be achieved through active listening and strengthening of young people’s voices [41]. Furthermore, developing an ethos of mutual respect and facilitating open but considerate dialogue between young people and group facilitators will be prioritised [1, 38]. For example, shared working principles or ‘terms of reference’ will be established within the first PI&E session. This focus will help to create a secure, mutually respectful, environment for all and set out group expectations for negotiating working together.

### Facilitating transparency

A lack of communication or evidence that public contributors’ suggestions have been taken on board has been identified as a source of disengagement and disappointment [38]. To mitigate this, the researchers will establish an open feedback dialogue with advisors. For example, the lead researcher (LC) will log reflections following each PI&E session. These reflections will be converted into a summary and shared alongside a ‘you said-we did’ style document at the start of the following session. This will help build momentum, evidence impact, and offer a further opportunity to discuss and amend any misrepresentation of ideas. Should a suggestion fall outside the scope of the project, this will be clearly communicated with group members and an alternative, workable, compromise established. This will help to ensure proportionality and manage expectations of contributors [23, 42].

Furthermore, we will also work to build trust and transparency with the host school. As sessions are delivered in a school setting, it is important that senior educational staff are happy with their content. The researchers will therefore maintain an open dialogue with schools about the nature of sessions. This will be achieved through the production and sharing of session plans. These will be produced in a similar format to lesson plans commonly used by teachers to enhance familiarity and understanding. All session plans will be shared in advance of each session, providing the opportunity to comment, adjust and amend. A senior staff member will also be sent an anonymised summary of key points following the session.

### Supplementary PI&E

Not all school-aged young people will feel comfortable or willing to engage with conversations surrounding health research within the context of a school setting. Students who are disengaged from school are more likely to have a complex risk profile, unmet-need, and poorer health status [43]. This makes them an incredibly important population to engage with, particularly given the context of this present research. Therefore, one or two stand-alone PI&E workshops will be designed to take place within a separate Youth Theatre setting. These will be conducted locally, working with an East of England youth theatre company, with all members invited to participate. The workshops will explore key concepts relevant to the research questions, however the specific content will be developed in conversation with young people and in response to project needs. To promote maximum engagement, additional advice will also be sought from youth theatre experts on best practice, logistics, and practicalities of conducting creative workshops with young people. Young people’s perspective will likely be captured and explored through the recording of a youth-led performance piece. Additional insights will be recorded within a nominated notetaker’s and/or the lead researcher’s (LC) field notes.

The perspective of school staff will also be captured. One or two educational professionals will help shape the project as peer-researchers from the development of this protocol, to interpretation and dissemination of results. Sessions will be held separately to those with young person advisors, although insights and perspectives may be anonymously shared across groups in order to explore experiences and opinions from different points of view. As with advisors, an open dialogue, sense of partnership, and needs-based training will be established. However, this will be more informal and in response to project need as opposed to a fixed and regular meeting schedule.

Lastly, supplementary attendance at advisory groups and additional ad-hoc PI&E activities may also be conducted as required. These PI&E activities will utilise existing and established networks/advisory groups. Sessions will aim to gather opinions from a different but meaningful perspective not already captured within scheduled PI&E. This may include parent populations, research staff, and/or those from specific underrepresented/clinical groups.

### Reviewing and adapting protocols

Evaluation is an essential component of learning and enhancement of PI&E [15, 16]. However, typically, evaluation is underreported [44] or falls to the end of PI&E [19]. This means opportunities to grow and implement change can be limited. Therefore, we will embed opportunities to evaluate and feedback throughout the project. This will be achieved through reflective ‘looking back’ group conversations surrounding what advisors and host schools like/dislike about the sessions. However, it is important to also listen and build upon this feedback. Within Thomas et al’s (2023) systematic review, this was highlighted to be a typical and valuable approach to assessing PI&E [8].

Impactful PI&E with young people relies upon responsive, tailored processes, driven by collaborators needs [8]. This protocol therefore further adopts an iterative approach. This means that adaptations may be made to the form, frequency, nature and location of PI&E activities. All decisions will be made carefully with young people, school staff, and the research team. Any disagreements will be discussed openly. Adaptions will be recorded within a dated decision log alongside a clear rationale for changes. We hope this approach will facilitate enjoyment, comfort, and improvement, whilst minimising participation barriers.

In addition, underpinned by the Public Involvement Impact Assessment Framework (PIIAF) [45] and Oliver et al’s framework [22] we will undertake an impact assessment of PI&E activities at the end of the project. This will not only consider the influence of PI&E on the research itself, but the impact on those involved. Consideration will also be given to the unique contexts to which the work was undertaken within.

### Dissemination

A bespoke dissemination strategy will be co-developed with advisors, tailored to enhance reach and access to both young people and school populations. In addition, we aim to share our findings with school-based public health researchers through publication in open-access, peer-reviewed academic journals. In order to enhance the clarity and consistency in the reporting of our PI&E activities, we will utilise the Guidance for Reporting Involvement of Patients and the Public-2 (GRIPP-2) checklist within academic journal outputs [46].

### Timings and costs

Both time and financial constraints have further been identified as a challenge for undertaking PI&E [1, 9]. Issues typically centre around tensions between funding constraints, research grant duration, and time required to recruit, implement, and evaluate PI&E. Thus, ensuring PI&E activities are carefully costed and embedded throughout the research cycle is of importance.

School-based sessions will take place during term time only. To minimise impact and disruption to learning, the exact timing will be developed and arranged in conversation with the host school. To further complement this schedule, the stand-alone theatre workshops will likely be conducted during school holidays. However, the schedule will remain flexible. Additional PI&E activities may be added and the timings updated.

All activities have been initially costed in accordance with NIHR Involve guidelines [15]. Due to the flexible and iterative nature of this protocol, a contingency fund has also been costed to facilitate necessary adaptions. This includes allowance for travel, subsidiaries, dissemination materials, and remuneration of public contributors in the form of vouchers. However, as students will be semi-regularly coming out of lessons to undertake PI&E activities, the host school highlighted the potential to cause harm and conflict amongst peers. Although contrary to guidance, remuneration in this context was deemed too great a risk. Donations to a charity of choice will be discussed as a possible alternative, however navigating university accounting processes are an anticipated challenge with this approach. This tension highlights the importance of local expertise, dialogue and nuanced approaches when developing PI&E protocols within school settings. For the host school, the learning potential, research experience, and opportunity to increase oracy, aspirations and confidence were of greater importance than financial compensation.

### Ethics

PI&E activities do not require approval from a research ethics committee. Therefore, formal ethical approval will not be sought. Nonetheless, Mitchell et al. (2019) highlight that complex ethical issues can arise throughout PI&E with young people [47]. Due to the population and potentially sensitive nature of this PI&E, steps to safeguard collaborators will be taken. Any ethical issues which arise during the project will also be carefully recorded and reported.

To reduce harms, safeguarding protocols and reporting procedures of the host school/theatre company will be followed. This includes securing an enhanced Disclosure and Barring Service check for the lead researcher (LC) and following standard operating procedure if a disclosure is made. Given the context of some group members having lived experiences of physical and or mental health conditions this may be likely. As a former teacher the lead researcher (LC) is well placed to sensitively navigate, identify and report any safeguarding concerns. This is because she will likely have a stronger understanding of safeguarding nuances than a doctoral student/researcher with less exposure to young people/school settings. LC also has previous experience conducting PI&E within school settings (for example Grant et al., 2020 [48].

In addition, where sessions are anticipated to explore sensitive topics, advisors will be sign-posted to carefully selected, age-appropriate materials. These resources will initially be guided by expertise within the research team. They will include resources which are available 24/7, free to access, and targeted at young people. Whilst young people will not initially select the resources, their appropriacy and accessibility will be raised and discussed within advisory sessions, with any adjustments made. Furthermore, utilising the co-established ‘terms of reference’ will help advisors navigate, process, and prepare for any potential disclosures of group members.

Permission to initially participate will be sought from young people and (via an opt-out methodology) parents/caregivers. An opt-out methodology can help promote inclusivity in health research [49] and is a model commonly adopted within school settings. However, additional written parental consent and individual verbal assent will be also sought from individuals prior to publication of individual names within any future outputs.

Advisors will routinely be reminded of their right to withdraw from PI&E sessions, and that they can do this without providing a reason.

## Discussion

This protocol draws on national guidance, evidence-informed frameworks, lessons learned from previous researchers, and advice from experts within the field. However, gold standard approaches within the specific context of this project have yet to be established. Schools are complex eco-systems, health an important yet sensitive topic, and young people a potentially vulnerable population. Therefore, there are anticipated practical and operational risks associated with implementing these PI&E protocols.

### Potential risks & risk management

For details of risks, likelihood of impact (low/moderate/high), and proposed management plans please see Table 1 on the following page.

**Table 1:** Discussion of potential risks and risk mitigation strategies

Description of risk	Level of risk	Risk management activities
**Young person attrition**	**HIGH**	• Where possible, sessions will be delivered in an engaging and accessible format. • PI&E activities will be developed and adapted in line with young people’s preferences. • Should a young person wish to stop/can no longer participate then a new young person will be recruited and trained.
**School attrition**	**MODERATE**	• The research team will be transparent in all communication with host school staff, seeking necessary permissions. • Operational preferences of how best to conduct PIE sessions will be discussed during initial conversations with senior leadership. • The lead researcher (LC) will offer to take part in activities to increase the engagement of the school. This may include participating in careers talks and/or leading assemblies.
**Knowledge barriers**	**HIGH**	• Training activities will be conducted on a needs-basis.
**Conflict of opinion**: conflict may arise between group members and/or the researcher and young people	**HIGH**	• An ethos of mutual respect and safe space for open communication will be established. • The lead researcher (LC) will remain transparent about decision making processes, carefully managing expectations about research constraints.
**Distress**: sensitive topics will likely be discussed during PIE activities which may be triggering, or risk young people becoming upset.	**HIGH**	• As above, a safe and respecting environment will be created. • A ‘trigger warning’ will be provided if discussion of a sensitive topic is planned. • Young people will be sign-posted to carefully selected resources and offered an opportunity to debrief.
**Safeguarding**: concern or disclosure made	**MODERATE**	• School standardised operating protocols will be followed in the event of a safeguarding disclosure. This involves LC reporting any concerns promptly and directly to a member of Senior Leadership (e.g. DB) or a designated safeguarding lead. • Terms of reference will help advisors navigate and process disclosures within the group should they occur.
**Misrepresentation**: there is scope for the researcher to misrepresent/ communicate the intentions/ experiences of advisors	**MODERATE**	• An open dialogue between researcher and advisor will be established, with the opportunity for reflection and feedback. • Young people will be provided an opportunity to comment on all main findings.

### Anticipated strengths and limitations

A strength of this protocol is the use of existing guidance, evidence and frameworks. However, evidence and approaches are typically developed with adult populations and community/clinical spaces in mind. Thus, guidance may not directly translate into a school-based health research context. We hope that working flexibly and mindfully with both schools and students may mitigate this risk and help us to navigate challenges which may arise.

Furthermore, developing meaningful partnerships takes time. Therefore, we anticipate that greater time investment will be required, with the research likely taking longer to complete. Careful time management and communication will therefore be essential to successful completion of protocols.

## Conclusion

Involving and engaging young people in school-based research has the potential to enhance the research we produce. However, implementing meaningful PI&E within school-settings is likely to be accompanied with challenges. We hope that our approach will help establish a successful and equal research partnership, and ultimately benefit all collaborators, the research, and those who may be impacted by the findings of our research.

## Data Availability

No datasets were generated or analysed during the current study.

## Abbreviations

NIHR: National Institute of Health and care Research
PI&E: Public Involvement and Engagement
SEN: Special Educational Need
SPHR: School for Public Health Research
